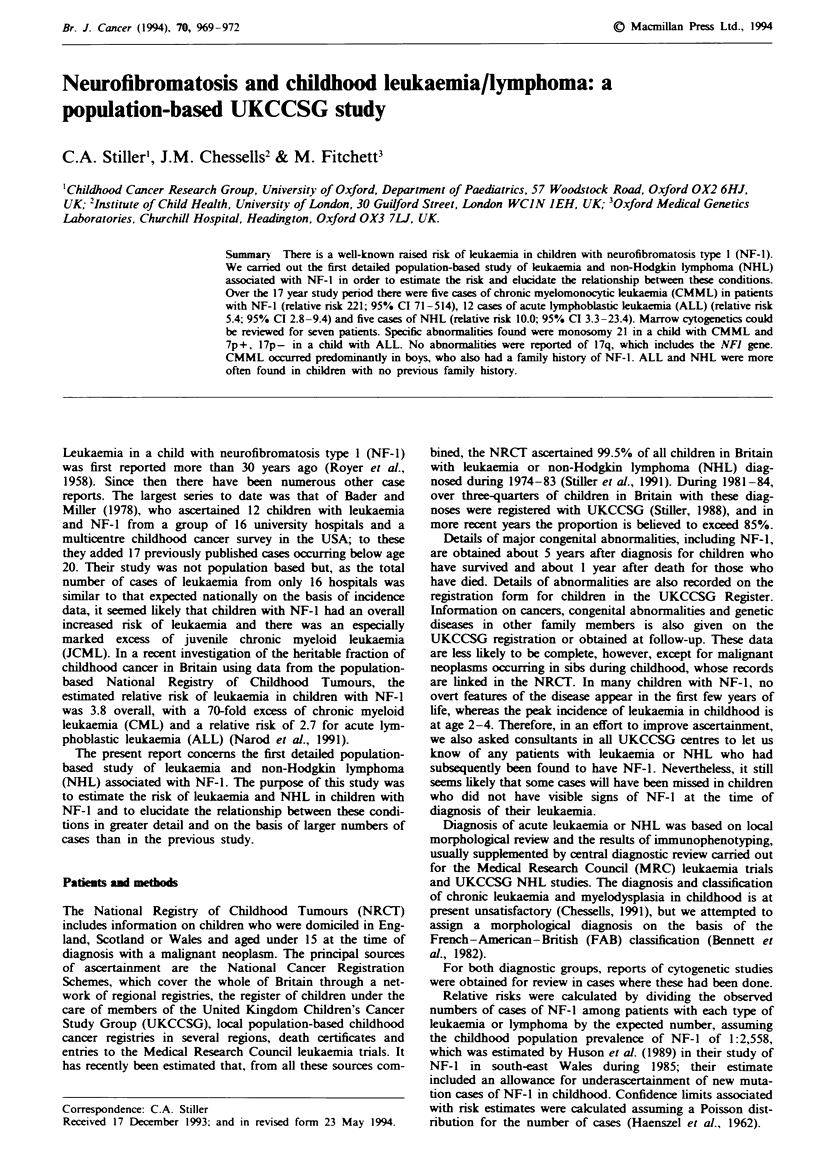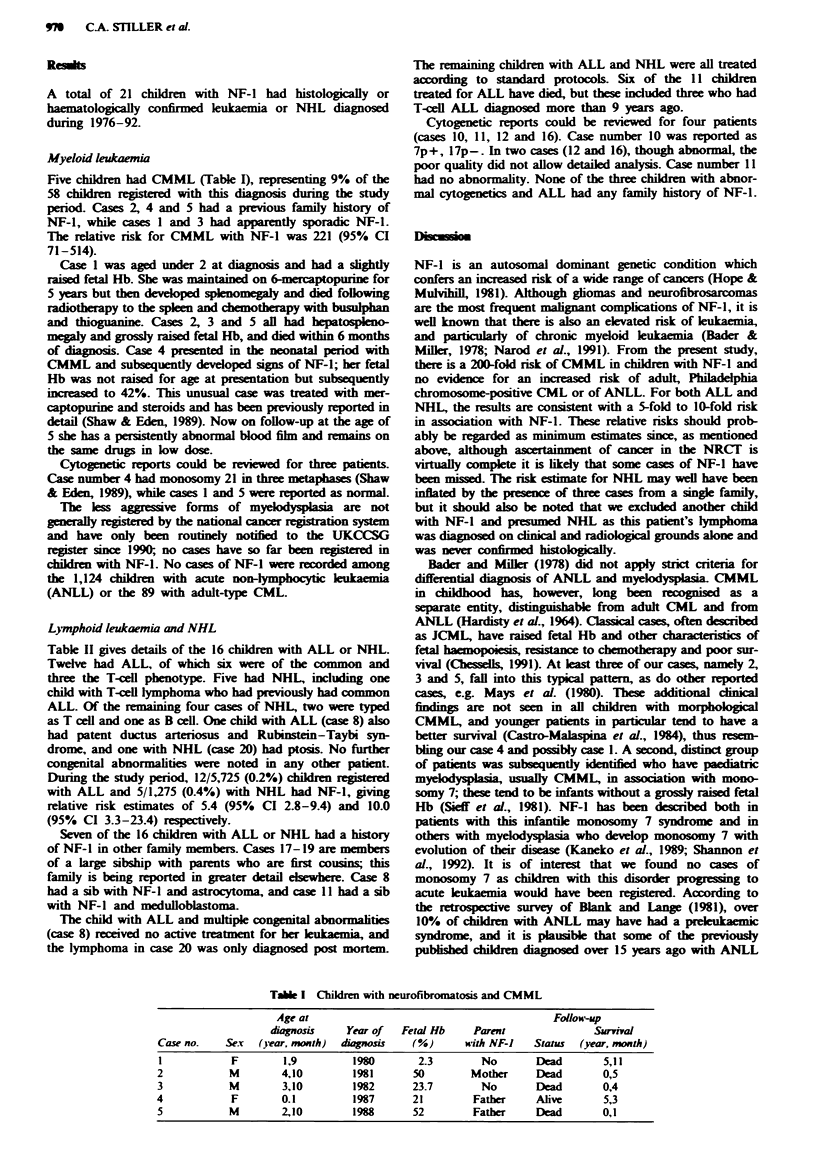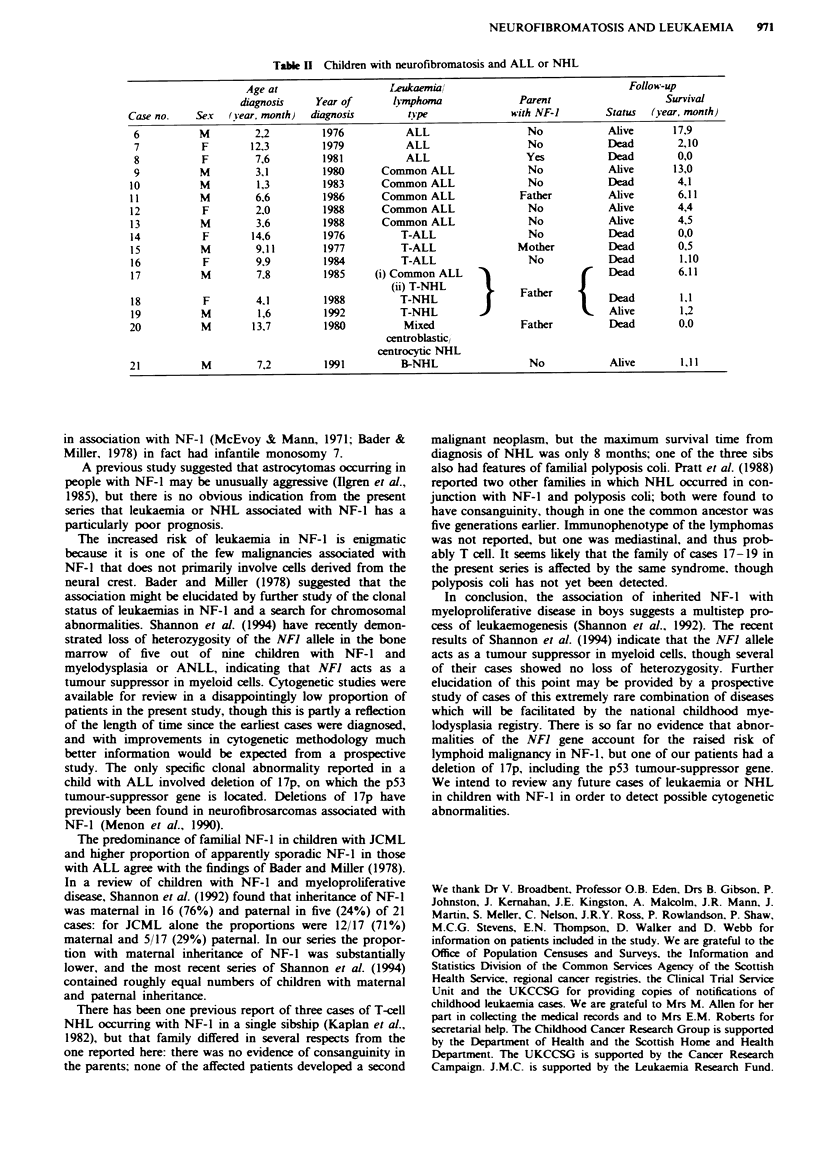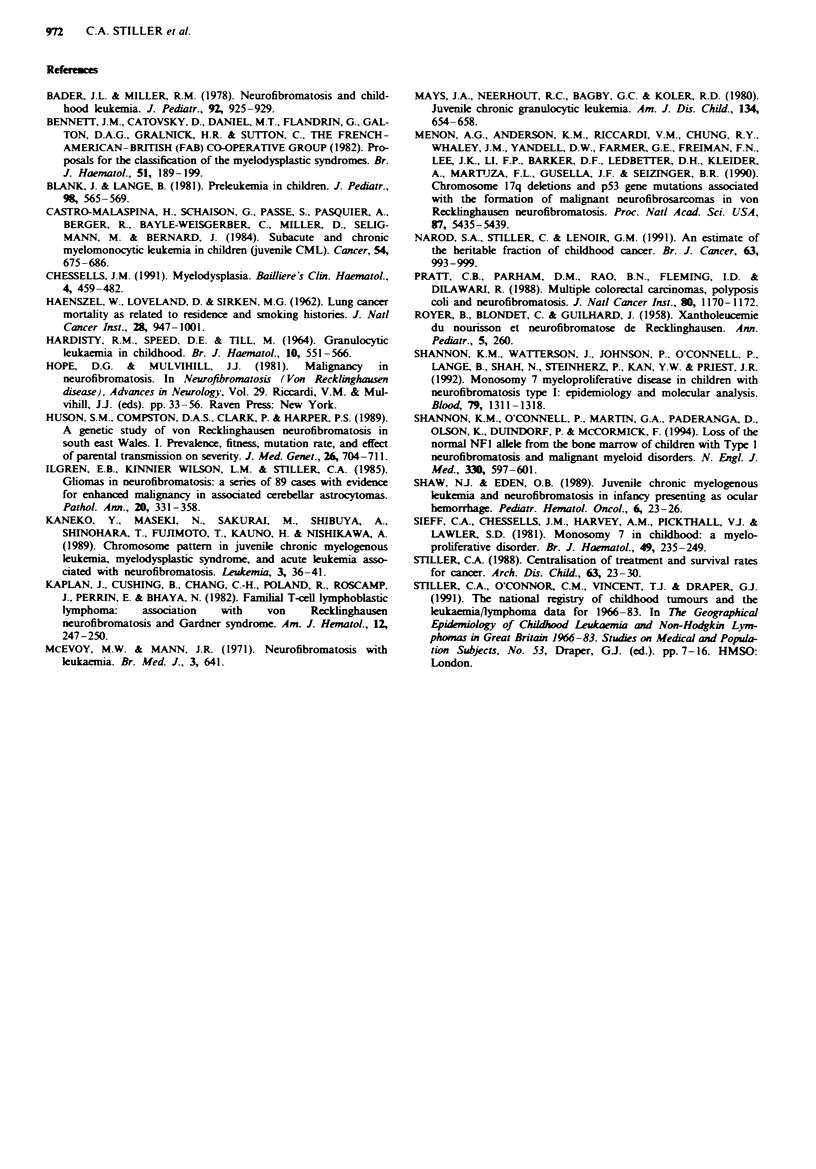# Neurofibromatosis and childhood leukaemia/lymphoma: a population-based UKCCSG study.

**DOI:** 10.1038/bjc.1994.431

**Published:** 1994-11

**Authors:** C. A. Stiller, J. M. Chessells, M. Fitchett

**Affiliations:** University of Oxford, Department of Paediatrics, UK.

## Abstract

There is a well-known raised risk of leukaemia in children with neurofibromatosis type 1 (NF-1). We carried out the first detailed population-based study of leukaemia and non-Hodgkin lymphoma (NHL) associated with NF-1 in order to estimate the risk and elucidate the relationship between these conditions. Over the 17 year study period there were five cases of chronic myelomonocytic leukaemia (CMML) in patients with NF-1 (relative risk 221; 95% CI 71-514), 12 cases of acute lymphoblastic leukaemia (ALL) (relative risk 5.4; 95% CI 2.8-9.4) and five cases of NHL (relative risk 10.0; 95% CI 3.3-23.4). Marrow cytogenetics could be reviewed for seven patients. Specific abnormalities found were monosomy 21 in a child with CMML and 7p+, 17p- in a child with ALL. No abnormalities were reported of 17q, which includes the NF1 gene. CMML occurred predominantly in boys, who also had a family history of NF-1. ALL and NHL were more often found in children with no previous family history.


Br. J. Cancer (1994), 70, 969-972 © Macmillan Press Ltd., 1994

### Neurofibromatosis and childhood leukaemia/lymphoma: a population-based UKCCSG study

C.A. Stiller[1], J.M. Chessells[2] & M. Fitchett[3]

[1]*Childhood Cancer Research Group, University of Oxford, Department of Paediatrics, 57 Woodstock Road, Oxford OX2 6HJ, UK;* [2]*Institute of Child Health, University of London, 30 Guilford Street, London WC1N 1EH, UK;* [3]*Oxford Medical Genetics Laboratories, Churchill Hospital, Headington, Oxford OX3 7LJ, UK.*

**Summary** There is a well-known raised risk of leukaemia in children with neurofibromatosis type 1 (NF-1). We carried out the first detailed population-based study of leukaemia and non-Hodgkin lymphoma (NHL) associated with NF-1 in order to estimate the risk and elucidate the relationship between these conditions. Over the 17 year study period there were five cases of chronic myelomonocytic leukaemia (CMML) in patients with NF-1 (relative risk 221; 95% CI 71-514), 12 cases of acute lymphoblastic leukaemia (ALL) (relative risk 5.4; 95% CI 2.8-9.4) and five cases of NHL (relative risk 10.0; 95% CI 3.3-23.4). Marrow cytogenetics could be reviewed for seven patients. Specific abnormalities found were monosomy 21 in a child with CMML and 7p+, 17p− in a child with ALL. No abnormalities were reported of 17q, which includes the *NF1* gene. CMML occurred predominantly in boys, who also had a family history of NF-1. ALL and NHL were more often found in children with no previous family history.

Leukaemia in a child with neurofibromatosis type 1 (NF-1) was first reported more than 30 years ago (Royer *et al.*, 1958). Since then there have been numerous other case reports. The largest series to date was that of Bader and Miller (1978), who ascertained 12 children with leukaemia and NF-1 from a group of 16 university hospitals and a multicentre childhood cancer survey in the USA; to these they added 17 previously published cases occurring below age 20. Their study was not population based but, as the total number of cases of leukaemia from only 16 hospitals was similar to that expected nationally on the basis of incidence data, it seemed likely that children with NF-1 had an overall increased risk of leukaemia and there was an especially marked excess of juvenile chronic myeloid leukaemia (JCML). In a recent investigation of the heritable fraction of childhood cancer in Britain using data from the population-based National Registry of Childhood Tumours, the estimated relative risk of leukaemia in children with NF-1 was 3.8 overall, with a 70-fold excess of chronic myeloid leukaemia (CML) and a relative risk of 2.7 for acute lymphoblastic leukaemia (ALL) (Narod *et al.*, 1991).

The present report concerns the first detailed population-based study of leukaemia and non-Hodgkin lymphoma (NHL) associated with NF-1. The purpose of this study was to estimate the risk of leukaemia and NHL in children with NF-1 and to elucidate the relationship between these conditions in greater detail and on the basis of larger numbers of cases than in the previous study.

### Patients and methods

The National Registry of Childhood Tumours (NRCT) includes information on children who were domiciled in England, Scotland or Wales and aged under 15 at the time of diagnosis with a malignant neoplasm. The principal sources of ascertainment are the National Cancer Registration Schemes, which cover the whole of Britain through a network of regional registries, the register of children under the care of members of the United Kingdom Children's Cancer Study Group (UKCCSG), local population-based childhood cancer registries in several regions, death certificates and entries to the Medical Research Council leukaemia trials. It has recently been estimated that, from all these sources combined, the NRCT ascertained 99.5% of all children in Britain with leukaemia or non-Hodgkin lymphoma (NHL) diagnosed during 1974-83 (Stiller *et al.*, 1991). During 1981-84, over three-quarters of children in Britain with these diagnoses were registered with UKCCSG (Stiller, 1988), and in more recent years the proportion is believed to exceed 85%.

Details of major congenital abnormalities, including NF-1, are obtained about 5 years after diagnosis for children who have survived and about 1 year after death for those who have died. Details of abnormalities are also recorded on the registration form for children in the UKCCSG Register. Information on cancers, congenital abnormalities and genetic diseases in other family members is also given on the UKCCSG registration or obtained at follow-up. These data are less likely to be complete, however, except for malignant neoplasms occurring in sibs during childhood, whose records are linked in the NRCT. In many children with NF-1, no overt features of the disease appear in the first few years of life, whereas the peak incidence of leukaemia in childhood is at age 2-4. Therefore, in an effort to improve ascertainment, we also asked consultants in all UKCCSG centres to let us know of any patients with leukaemia or NHL who had subsequently been found to have NF-1. Nevertheless, it still seems likely that some cases will have been missed in children who did not have visible signs of NF-1 at the time of diagnosis of their leukaemia.

Diagnosis of acute leukaemia or NHL was based on local morphological review and the results of immunophenotyping, usually supplemented by central diagnostic review carried out for the Medical Research Council (MRC) leukaemia trials and UKCCSG NHL studies. The diagnosis and classification of chronic leukaemia and myelodysplasia in childhood is at present unsatisfactory (Chessells, 1991), but we attempted to assign a morphological diagnosis on the basis of the French-American-British (FAB) classification (Bennett *et al.*, 1982).

For both diagnostic groups, reports of cytogenetic studies were obtained for review in cases where these had been done.

Relative risks were calculated by dividing the observed numbers of cases of NF-1 among patients with each type of leukaemia or lymphoma by the expected number, assuming the childhood population prevalence of NF-1 of 1:2,558, which was estimated by Huson *et al.* (1989) in their study of NF-1 in south-east Wales during 1985; their estimate included an allowance for underascertainment of new mutation cases of NF-1 in childhood. Confidence limits associated with risk estimates were calculated assuming a Poisson distribution for the number of cases (Haenszel *et al.*, 1962).

Correspondence: C.A. Stiller
Received 17 December 1993; and in revised form 23 May 1994.

### Results

A total of 21 children with NF-1 had histologically or haematologically confirmed leukaemia or NHL diagnosed during 1976–92.

### *Myeloid leukaemia*

Five children had CMML (Table I), representing 9% of the 58 children registered with this diagnosis during the study period. Cases 2, 4 and 5 had a previous family history of NF-1, while cases 1 and 3 had apparently sporadic NF-1. The relative risk for CMML with NF-1 was 221 (95% CI 71–514).

Case 1 was aged under 2 at diagnosis and had a slightly raised fetal Hb. She was maintained on 6-mercaptopurine for 5 years but then developed splenomegaly and died following radiotherapy to the spleen and chemotherapy with busulphan and thioguanine. Cases 2, 3 and 5 all had hepatosplenomegaly and grossly raised fetal Hb, and died within 6 months of diagnosis. Case 4 presented in the neonatal period with CMML and subsequently developed signs of NF-1; her fetal Hb was not raised for age at presentation but subsequently increased to 42%. This unusual case was treated with mercaptopurine and steroids and has been previously reported in detail (Shaw & Eden, 1989). Now on follow-up at the age of 5 she has a persistently abnormal blood film and remains on the same drugs in low dose.

Cytogenetic reports could be reviewed for three patients. Case number 4 had monosomy 21 in three metaphases (Shaw & Eden, 1989), while cases 1 and 5 were reported as normal.

The less aggressive forms of myelodysplasia are not generally registered by the national cancer registration system and have only been routinely notified to the UKCCSG register since 1990; no cases have so far been registered in children with NF-1. No cases of NF-1 were recorded among the 1,124 children with acute non-lymphocytic leukaemia (ANLL) or the 89 with adult-type CML.

### *Lymphoid leukaemia and NHL*

Table II gives details of the 16 children with ALL or NHL. Twelve had ALL, of which six were of the common and three the T-cell phenotype. Five had NHL, including one child with T-cell lymphoma who had previously had common ALL. Of the remaining four cases of NHL, two were typed as T cell and one as B cell. One child with ALL (case 8) also had patent ductus arteriosus and Rubinstein–Taybi syndrome, and one with NHL (case 20) had ptosis. No further congenital abnormalities were noted in any other patient. During the study period, 12/5,725 (0.2%) children registered with ALL and 5/1,275 (0.4%) with NHL had NF-1, giving relative risk estimates of 5.4 (95% CI 2.8–9.4) and 10.0 (95% CI 3.3–23.4) respectively.

Seven of the 16 children with ALL or NHL had a history of NF-1 in other family members. Cases 17–19 are members of a large sibship with parents who are first cousins; this family is being reported in greater detail elsewhere. Case 8 had a sib with NF-1 and astrocytoma, and case 11 had a sib with NF-1 and medulloblastoma.

The child with ALL and multiple congenital abnormalities (case 8) received no active treatment for her leukaemia, and the lymphoma in case 20 was only diagnosed post mortem. The remaining children with ALL and NHL were all treated according to standard protocols. Six of the 11 children treated for ALL have died, but these included three who had T-cell ALL diagnosed more than 9 years ago.

Cytogenetic reports could be reviewed for four patients (cases 10, 11, 12 and 16). Case number 10 was reported as 7p+, 17p−. In two cases (12 and 16), though abnormal, the poor quality did not allow detailed analysis. Case number 11 had no abnormality. None of the three children with abnormal cytogenetics and ALL had any family history of NF-1.

### Discussion

NF-1 is an autosomal dominant genetic condition which confers an increased risk of a wide range of cancers (Hope & Mulvihill, 1981). Although gliomas and neurofibrosarcomas are the most frequent malignant complications of NF-1, it is well known that there is also an elevated risk of leukaemia, and particularly of chronic myeloid leukaemia (Bader & Miller, 1978; Narod *et al.*, 1991). From the present study, there is a 200-fold risk of CMML in children with NF-1 and no evidence for an increased risk of adult, Philadelphia chromosome-positive CML or of ANLL. For both ALL and NHL, the results are consistent with a 5-fold to 10-fold risk in association with NF-1. These relative risks should probably be regarded as minimum estimates since, as mentioned above, although ascertainment of cancer in the NRCT is virtually complete it is likely that some cases of NF-1 have been missed. The risk estimate for NHL may well have been inflated by the presence of three cases from a single family, but it should also be noted that we excluded another child with NF-1 and presumed NHL as this patient's lymphoma was diagnosed on clinical and radiological grounds alone and was never confirmed histologically.

Bader and Miller (1978) did not apply strict criteria for differential diagnosis of ANLL and myelodysplasia. CMML in childhood has, however, long been recognised as a separate entity, distinguishable from adult CML and from ANLL (Hardisty *et al.*, 1964). Classical cases, often described as JCML, have raised fetal Hb and other characteristics of fetal haemopoiesis, resistance to chemotherapy and poor survival (Chessells, 1991). At least three of our cases, namely 2, 3 and 5, fall into this typical pattern, as do other reported cases, e.g. Mays *et al.* (1980). These additional clinical findings are not seen in all children with morphological CMML, and younger patients in particular tend to have a better survival (Castro-Malaspina *et al.*, 1984), thus resembling our case 4 and possibly case 1. A second, distinct group of patients was subsequently identified who have paediatric myelodysplasia, usually CMML, in association with monosomy 7; these tend to be infants without a grossly raised fetal Hb (Sieff *et al.*, 1981). NF-1 has been described both in patients with this infantile monosomy 7 syndrome and in others with myelodysplasia who develop monosomy 7 with evolution of their disease (Kaneko *et al.*, 1989; Shannon *et al.*, 1992). It is of interest that we found no cases of monosomy 7 as children with this disorder progressing to acute leukaemia would have been registered. According to the retrospective survey of Blank and Lange (1981), over 10% of children with ANLL may have had a preleukaemic syndrome, and it is plausible that some of the previously published children diagnosed over 15 years ago with ANLL

**Table I** Children with neurofibromatosis and CMML

| Case no. | Sex | Age at diagnosis (year, month) | Year of diagnosis | Fetal Hb (%) | Parent with NF-1 | Follow-up: Status | Follow-up: Survival (year, month) |
|---|---|---|---|---|---|---|---|
| 1 | F | 1,9 | 1980 | 2.3 | No | Dead | 5,11 |
| 2 | M | 4,10 | 1981 | 50 | Mother | Dead | 0,5 |
| 3 | M | 3,10 | 1982 | 23.7 | No | Dead | 0,4 |
| 4 | F | 0.1 | 1987 | 21 | Father | Alive | 5,3 |
| 5 | M | 2,10 | 1988 | 52 | Father | Dead | 0,1 |

**Table II** Children with neurofibromatosis and ALL or NHL

| Case no. | Sex | Age at diagnosis (year, month) | Year of diagnosis | Leukaemia/ lymphoma type | Parent with NF-1 | Follow-up Status | Follow-up Survival (year, month) |
|---|---|---|---|---|---|---|---|
| 6 | M | 2,2 | 1976 | ALL | No | Alive | 17,9 |
| 7 | F | 12,3 | 1979 | ALL | No | Dead | 2,10 |
| 8 | F | 7,6 | 1981 | ALL | Yes | Dead | 0,0 |
| 9 | M | 3,1 | 1980 | Common ALL | No | Alive | 13,0 |
| 10 | M | 1,3 | 1983 | Common ALL | No | Dead | 4,1 |
| 11 | M | 6,6 | 1986 | Common ALL | Father | Alive | 6,11 |
| 12 | F | 2,0 | 1988 | Common ALL | No | Alive | 4,4 |
| 13 | M | 3,6 | 1988 | Common ALL | No | Alive | 4,5 |
| 14 | F | 14,6 | 1976 | T-ALL | No | Dead | 0,0 |
| 15 | M | 9,11 | 1977 | T-ALL | Mother | Dead | 0,5 |
| 16 | F | 9,9 | 1984 | T-ALL | No | Dead | 1,10 |
| 17 | M | 7,8 | 1985 | (i) Common ALL (ii) T-NHL | } | { Dead | 6,11 |
| 18 | F | 4,1 | 1988 | T-NHL | Father (cases 17–19) | Dead | 1,1 |
| 19 | M | 1,6 | 1992 | T-NHL | } | Alive | 1,2 |
| 20 | M | 13,7 | 1980 | Mixed centroblastic/ centrocytic NHL | Father | Dead | 0,0 |
| 21 | M | 7,2 | 1991 | B-NHL | No | Alive | 1,11 |

in association with NF-1 (McEvoy & Mann, 1971; Bader & Miller, 1978) in fact had infantile monosomy 7.

A previous study suggested that astrocytomas occurring in people with NF-1 may be unusually aggressive (Ilgren *et al.*, 1985), but there is no obvious indication from the present series that leukaemia or NHL associated with NF-1 has a particularly poor prognosis.

The increased risk of leukaemia in NF-1 is enigmatic because it is one of the few malignancies associated with NF-1 that does not primarily involve cells derived from the neural crest. Bader and Miller (1978) suggested that the association might be elucidated by further study of the clonal status of leukaemias in NF-1 and a search for chromosomal abnormalities. Shannon *et al.* (1994) have recently demonstrated loss of heterozygosity of the *NF1* allele in the bone marrow of five out of nine children with NF-1 and myelodysplasia or ANLL, indicating that *NF1* acts as a tumour suppressor in myeloid cells. Cytogenetic studies were available for review in a disappointingly low proportion of patients in the present study, though this is partly a reflection of the length of time since the earliest cases were diagnosed, and with improvements in cytogenetic methodology much better information would be expected from a prospective study. The only specific clonal abnormality reported in a child with ALL involved deletion of 17p, on which the p53 tumour-suppressor gene is located. Deletions of 17p have previously been found in neurofibrosarcomas associated with NF-1 (Menon *et al.*, 1990).

The predominance of familial NF-1 in children with JCML and higher proportion of apparently sporadic NF-1 in those with ALL agree with the findings of Bader and Miller (1978). In a review of children with NF-1 and myeloproliferative disease, Shannon *et al.* (1992) found that inheritance of NF-1 was maternal in 16 (76%) and paternal in five (24%) of 21 cases: for JCML alone the proportions were 12/17 (71%) maternal and 5/17 (29%) paternal. In our series the proportion with maternal inheritance of NF-1 was substantially lower, and the most recent series of Shannon *et al.* (1994) contained roughly equal numbers of children with maternal and paternal inheritance.

There has been one previous report of three cases of T-cell NHL occurring with NF-1 in a single sibship (Kaplan *et al.*, 1982), but that family differed in several respects from the one reported here: there was no evidence of consanguinity in the parents; none of the affected patients developed a second malignant neoplasm, but the maximum survival time from diagnosis of NHL was only 8 months; one of the three sibs also had features of familial polyposis coli. Pratt *et al.* (1988) reported two other families in which NHL occurred in conjunction with NF-1 and polyposis coli; both were found to have consanguinity, though in one the common ancestor was five generations earlier. Immunophenotype of the lymphomas was not reported, but one was mediastinal, and thus probably T cell. It seems likely that the family of cases 17–19 in the present series is affected by the same syndrome, though polyposis coli has not yet been detected.

In conclusion, the association of inherited NF-1 with myeloproliferative disease in boys suggests a multistep process of leukaemogenesis (Shannon *et al.*, 1992). The recent results of Shannon *et al.* (1994) indicate that the *NF1* allele acts as a tumour suppressor in myeloid cells, though several of their cases showed no loss of heterozygosity. Further elucidation of this point may be provided by a prospective study of cases of this extremely rare combination of diseases which will be facilitated by the national childhood myelodysplasia registry. There is so far no evidence that abnormalities of the *NF1* gene account for the raised risk of lymphoid malignancy in NF-1, but one of our patients had a deletion of 17p, including the p53 tumour-suppressor gene. We intend to review any future cases of leukaemia or NHL in children with NF-1 in order to detect possible cytogenetic abnormalities.

We thank Dr V. Broadbent, Professor O.B. Eden, Drs B. Gibson, P. Johnston, J. Kernahan, J.E. Kingston, A. Malcolm, J.R. Mann, J. Martin, S. Meller, C. Nelson, J.R.Y. Ross, P. Rowlandson, P. Shaw, M.C.G. Stevens, E.N. Thompson, D. Walker and D. Webb for information on patients included in the study. We are grateful to the Office of Population Censuses and Surveys, the Information and Statistics Division of the Common Services Agency of the Scottish Health Service, regional cancer registries, the Clinical Trial Service Unit and the UKCCSG for providing copies of notifications of childhood leukaemia cases. We are grateful to Mrs M. Allen for her part in collecting the medical records and to Mrs E.M. Roberts for secretarial help. The Childhood Cancer Research Group is supported by the Department of Health and the Scottish Home and Health Department. The UKCCSG is supported by the Cancer Research Campaign. J.M.C. is supported by the Leukaemia Research Fund.

### References

BADER, J.L. & MILLER, R.M. (1978). Neurofibromatosis and childhood leukemia. *J. Pediatr.*, **92**, 925-929.

BENNETT, J.M., CATOVSKY, D., DANIEL, M.T., FLANDRIN, G., GALTON, D.A.G., GRALNICK, H.R. & SUTTON, C., THE FRENCH-AMERICAN-BRITISH (FAB) CO-OPERATIVE GROUP (1982). Proposals for the classification of the myelodysplastic syndromes. *Br. J. Haematol.*, **51**, 189-199.

BLANK, J. & LANGE, B. (1981). Preleukemia in children. *J. Pediatr.*, **98**, 565-569.

CASTRO-MALASPINA, H., SCHAISON, G., PASSE, S., PASQUIER, A., BERGER, R., BAYLE-WEISGERBER, C., MILLER, D., SELIGMANN, M. & BERNARD, J. (1984). Subacute and chronic myelomonocytic leukemia in children (juvenile CML). *Cancer*, **54**, 675-686.

CHESSELLS, J.M. (1991). Myelodysplasia. *Bailliere's Clin. Haematol.*, **4**, 459-482.

HAENSZEL, W., LOVELAND, D. & SIRKEN, M.G. (1962). Lung cancer mortality as related to residence and smoking histories. *J. Natl Cancer Inst.*, **28**, 947-1001.

HARDISTY, R.M., SPEED, D.E. & TILL, M. (1964). Granulocytic leukaemia in childhood. *Br. J. Haematol.*, **10**, 551-566.

HOPE, D.G. & MULVIHILL, J.J. (1981). Malignancy in neurofibromatosis. In *Neurofibromatosis (Von Recklinghausen disease), Advances in Neurology*, Vol. 29. Riccardi, V.M. & Mulvihill, J.J. (eds). pp. 33-56. Raven Press: New York.

HUSON, S.M., COMPSTON, D.A.S., CLARK, P. & HARPER, P.S. (1989). A genetic study of von Recklinghausen neurofibromatosis in south east Wales. I. Prevalence, fitness, mutation rate, and effect of parental transmission on severity. *J. Med. Genet.*, **26**, 704-711.

ILGREN, E.B., KINNIER WILSON, L.M. & STILLER, C.A. (1985). Gliomas in neurofibromatosis: a series of 89 cases with evidence for enhanced malignancy in associated cerebellar astrocytomas. *Pathol. Ann.*, **20**, 331-358.

KANEKO, Y., MASEKI, N., SAKURAI, M., SHIBUYA, A., SHINOHARA, T., FUJIMOTO, T., KAUNO, H. & NISHIKAWA, A. (1989). Chromosome pattern in juvenile chronic myelogenous leukemia, myelodysplastic syndrome, and acute leukemia associated with neurofibromatosis. *Leukemia*, **3**, 36-41.

KAPLAN, J., CUSHING, B., CHANG, C.-H., POLAND, R., ROSCAMP, J., PERRIN, E. & BHAYA, N. (1982). Familial T-cell lymphoblastic lymphoma: association with von Recklinghausen neurofibromatosis and Gardner syndrome. *Am. J. Hematol.*, **12**, 247-250.

MCEVOY, M.W. & MANN, J.R. (1971). Neurofibromatosis with leukaemia. *Br. Med. J.*, **3**, 641.

MAYS, J.A., NEERHOUT, R.C., BAGBY, G.C. & KOLER, R.D. (1980). Juvenile chronic granulocytic leukemia. *Am. J. Dis. Child.*, **134**, 654-658.

MENON, A.G., ANDERSON, K.M., RICCARDI, V.M., CHUNG, R.Y., WHALEY, J.M., YANDELL, D.W., FARMER, G.E., FREIMAN, F.N., LEE, J.K., LI, F.P., BARKER, D.F., LEDBETTER, D.H., KLEIDER, A., MARTUZA, F.L., GUSELLA, J.F. & SEIZINGER, B.R. (1990). Chromosome 17q deletions and p53 gene mutations associated with the formation of malignant neurofibrosarcomas in von Recklinghausen neurofibromatosis. *Proc. Natl Acad. Sci. USA*, **87**, 5435-5439.

NAROD, S.A., STILLER, C. & LENOIR, G.M. (1991). An estimate of the heritable fraction of childhood cancer. *Br. J. Cancer*, **63**, 993-999.

PRATT, C.B., PARHAM, D.M., RAO, B.N., FLEMING, I.D. & DILAWARI, R. (1988). Multiple colorectal carcinomas, polyposis coli and neurofibromatosis. *J. Natl Cancer Inst.*, **80**, 1170-1172.

ROYER, B., BLONDET, C. & GUILHARD, J. (1958). Xantholeucemie du nourisson et neurofibromatose de Recklinghausen. *Ann. Pediatr.*, **5**, 260.

SHANNON, K.M., WATTERSON, J., JOHNSON, P., O'CONNELL, P., LANGE, B., SHAH, N., STEINHERZ, P., KAN, Y.W. & PRIEST, J.R. (1992). Monosomy 7 myeloproliferative disease in children with neurofibromatosis type I: epidemiology and molecular analysis. *Blood*, **79**, 1311-1318.

SHANNON, K.M., O'CONNELL, P., MARTIN, G.A., PADERANGA, D., OLSON, K., DUINDORF, P. & MCCORMICK, F. (1994). Loss of the normal NF1 allele from the bone marrow of children with Type 1 neurofibromatosis and malignant myeloid disorders. *N. Engl. J. Med.*, **330**, 597-601.

SHAW, N.J. & EDEN, O.B. (1989). Juvenile chronic myelogenous leukemia and neurofibromatosis in infancy presenting as ocular hemorrhage. *Pediatr. Hematol. Oncol.*, **6**, 23-26.

SIEFF, C.A., CHESSELLS, J.M., HARVEY, A.M., PICKTHALL, V.J. & LAWLER, S.D. (1981). Monosomy 7 in childhood: a myeloproliferative disorder. *Br. J. Haematol.*, **49**, 235-249.

STILLER, C.A. (1988). Centralisation of treatment and survival rates for cancer. *Arch. Dis. Child.*, **63**, 23-30.

STILLER, C.A., O'CONNOR, C.M., VINCENT, T.J. & DRAPER, G.J. (1991). The national registry of childhood tumours and the leukaemia/lymphoma data for 1966-83. In *The Geographical Epidemiology of Childhood Leukaemia and Non-Hodgkin Lymphomas in Great Britain 1966-83. Studies on Medical and Population Subjects, No. 53*, Draper, G.J. (ed.). pp. 7-16. HMSO: London.